# Genetic Programming $\varvec{+}$ Proof Search $\varvec{=}$ Automatic Improvement

**DOI:** 10.1007/s10817-017-9409-5

**Published:** 2017-03-07

**Authors:** Zoltan A. Kocsis, Jerry Swan

**Affiliations:** Department of Computer Science, The University of York, Deramore Lane, York, YO10 5GH UK

**Keywords:** Program synthesis, Software maintenance, Search Based Software Engineering, Genetic Programming

## Abstract

Search Based Software Engineering techniques are emerging as important tools for software maintenance. Foremost among these is Genetic Improvement, which has historically applied the stochastic techniques of Genetic Programming to optimize pre-existing program code. Previous work in this area has not generally preserved program semantics and this article describes an alternative to the traditional mutation operators used, employing deterministic proof search in the sequent calculus to yield semantics-preserving transformations on algebraic data types. Two case studies are described, both of which are applicable to the recently-introduced ‘grow and graft’ technique of Genetic Improvement: the first extends the expressiveness of the ‘grafting’ phase and the second transforms the representation of a list data type to yield an asymptotic efficiency improvement.

## Introduction

The software development lifecycle has always been dominated by maintenance costs [1, 2] and the increasingly large scale of modern software systems means that greater automation of this maintenance burden is highly desirable. However, ‘round-trip’ software re-engineering tools are not currently adequate to transform large volumes of legacy code written in popular languages (e.g. C/C++, Java) into a form which is amenable to automated reasoning. Attention has therefore recently turned to the use of Search Based Software Engineering, an increasingly popular trend for the application of optimization techniques to problems in software engineering. In particular, Genetic Programming (GP) [3] is a stochastic approach to program generation driven by Darwinian principles of ‘survival of the fittest’, in which a collection of programs (the *population*) is iteratively improved via the nature-inspired operations of *recombination* and *mutation*. The transformation of pre-existing (and typically human-written) program source code via GP is known as Genetic Improvement (GI) [4, 5]. The majority of previous work in GI has not preserved program semantics, which we believe presents an obstacle to more widespread acceptance.

Our previous work on improvement of human-written programs that is semantics-preserving includes: a 10,000-fold speedup of database queries on terabyte datasets within the Apache Spark analytics framework [6]; automatic generation of an energy-efficient version of the Quicksort algorithm (consuming half the power of the popular ‘median of first, mid, last’ method due to Sedgewick) on pathological input distributions [7]; a 24% improvement in energy consumption by optimizing a single widely-used class in Google’s Guava collection library [8]; automatic repair of over 400 systematic errors in the implementation of the Apache Hadoop analytics framework whilst simultaneously significantly improving performance [9]; automated speedup of concurrent versions of divide-and-conquer algorithms (Quicksort, Strassen matrix multiplication and the FFT) [10]. However, each of these approaches require experiments to be explicitly framed (in a separate manner for each technique) in terms of semantics-preserving operations.

Type-directed approaches have the advantage of genericity and can provide stronger assurance of correctness for users of search based techniques. In addition, type information can in some cases replace the need for stochastic search altogether. In particular, it is applicable to the recently-popularized ‘grow and graft’ strategy [11] (in which code is synthesized in a toy environment and then transplanted into a target program) is intended to be usable by programmers with no deep knowledge of the target system. This cannot be achieved unless fruitful grafting points can be identified without human intervention. We show that our approach can address several shortcomings of ‘grow and graft’ by replacing the stochastic ‘graft’ phase with a deterministic proof search.

We proceed to describe a general approach to synthesis by exploiting the semantics derivable from a sufficiently expressive type system. In particular, we apply techniques from type theory to perform deterministic transformations of source code. Since the transformations we apply are semantics-preserving and their application is not strictly limited to Genetic Programming, we have adopted the term ‘Automatic Improvement Programming’ (AIP). We provide an overview of the theory underlying the implementation of our PolyFunic AIP system, which operates on programs in the Scala programming language [12]. Building upon the reflection facility of the language, PolyFunic can identify (a class of) data types and use deterministic search in the space of logical proofs to define transformations between them. The Scala source code for executing the transformations can then be generated from the proof trees via the Curry–Howard isomorphism [13].

### Overview

There is growing interest in automatic programming techniques for software maintenance and evolution. In addition to the work described in the previous section, one noted success has been a 70-fold improvement in runtime performance [14] for a 50,000 line C++ program in widespread use. In general, the optimisation criteria can include a variety of functional and non-functional properties [15]. This suggests that exploring the space of data types is likely to be useful, since this allows (for example) memory to be traded for execution speed. In GP in general, programs are typically represented as expressions trees, stack- or register-machines. In GI, the tendency is to use Abstract Syntax Trees (AST) or e.g. delta-edit sequences of ASTs [16]. The type system of a program can contain useful semantic information, which is of particularly value in a functional (referentially-transparent) context. In particular, types can give information about what a program *calculates*, i.e. a denotational semantics [17]. For example, for $f{:}\, List \rightarrow {\mathbb {N}}$, it can be determined from the type of *f* (via the mechanism of ‘free theorems’) that *f* is a function of the length of the list [18]. More expressive languages (e.g. Agda [19]) allow even stronger inference of the underlying semantics. For example, each of the 3 terms in the following expression are types, and the two numbers $a,b \in {\mathbb {N}}$ returned by the function 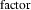 when given an argument $c \in {\mathbb {N}}$ are always guaranteed to satisfy the predicate $a * b == c$:

$$\begin{aligned} \mathrm {factor}{:}\,(c \in {\mathbb {N}} ) \rightarrow (a,b \in {\mathbb {N}}) \times (a * b == c) \end{aligned}$$

In subsequent sections, we describe a method of program transformation that operates on algebraic data types (ADTs) and which can be considered as a semantics-preserving mutation operator. Although algebraic data types share an acronym with *abstract* data types, they should not be confused with the latter notion. ADTs are ubiquitous in functional programming (indeed, they are the only data types in the Haskell language, for example) and with the increasing trend for incorporating aspects of functional programming into mainstream languages, it might be expected that ADTs will become more widely known. Although our implementation uses Scala, the technique is applicable to any language which can express ADTs, whether explicitly or by convention—in principle this could even be achieved in more traditional imperative languages such as C, but the idiosyncrasies of the type system and the lack of sophisticated reflection facilities would make the implementation a daunting prospect.

As described in more detail subsequently, ADTs are inductively-defined in terms of multiple type-constructors via choice, tupling and higher-order functions [20]. A simple example of an ADT is the singly-linked list, having two constructors, 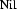 for the empty list and 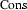 for combining an element with a pre-existing list to create a new list. In Scala this is expressed as 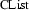 as shown in Listing 1. For those unfamiliar with Scala, a 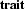 can be considered as equivalent to a Java™ interface and the 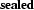 keyword ensures that the only subclasses which can exist are those given in the listing (the latter is necessary in order to guarantee the validity of the equational derivation described in Sect. 3). A 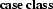 denotes a value (as opposed to reference) type that can appear in pattern-matching expressions.

The term ‘algebraic data type’ arises from the fact that ADTs have an ‘equational’ representation, viz. the notion of ‘choice of constructor’ corresponds to ‘$+$’, tupling of types corresponds to ‘$\times$’ and functions are denoted by exponentiation.
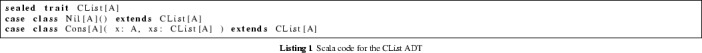


The transformation process is achieved via a search for a semantics-preserving mapping which replaces a designated source type with some alternative (but functionally compatible) target type. As detailed subsequently, the equational representations for source and target types can be automatically derived from the type declaration and then used to guide the search process. The search method is that of *deterministic proof search* [13], resulting in a proof that the source can be transformed into the target. This proof can then be turned into a program fragment which performs the required mapping via the Curry–Howard isomorphism (see [13], Ch. 3). Such a mapping is desirable for two reasons:

1. It can increase the number of useful graft operations available in the ‘graft’ phase of ‘grow and graft’ GI [11].
2. Being able to choose between functionally equivalent target types allows the trade-off between non-functional properties to be explored. In particular, it may be possible to choose a target type with asymptotically superior performance [21].

Case studies for both of these applications appear in Sect. 6. The former application is novel and the latter provides further detail to that given in [21]. The remainder of this article is as follows: Sect. 2 describes related work in both GI and formal methods of program synthesis, together with some of the open issues in GI. We describe the transformation process in Sects. 3 and 3.3 and detail the two case studies above in Sect. 6. Section 7 gives conclusions and avenues for future work.

## Related Work

### Genetic Improvement

As discussed above, GI is the application of Genetic Programming techniques [3] to (typically human-engineered) source or object code. Although in relative infancy, GI has been used to improve performance [14]; obtain multi-objective trade-offs between non-functional properties [22] and fix bugs [2]. Two prevalent examples of GI systems are the *GISMOE* system of Langdon and Harman [14] and *GenProg* by Le Goues et al. [2]. The *GISMOE* system is used to improve the performance of the 50,000 lines of C++ code of the widely-used DNA sequencing system Bowtie2. In [2], Le Goues et al. describe the latest version of their GenProg GI software for bug repair in ‘C’ programs. GenProg employs negative test cases for encoding the bug and a set of positive test cases describing behavioural invariants. In order to conserve memory, program variants are represented as a sequence of edit operations (‘patches’).

As might be expected of an emerging research topic, there are some issues with GI. The most notable issue is loss of correctness: it is common practice in GI to validate against a test suite (if one exists) and/or treat the seed program as an Oracle. This approach is of course subject to the caveat that “program testing can be used very effectively to show the presence of bugs but never to show their absence” [23]. Indeed, some results from more popular GI systems have recently been called into question. In a study by Qi et al. [24] of the validity of repairs generated by *GenProg* and the RSRepair [25] and AE [26] systems, it was determined that these systems actually failed to produce valid repairs on at least half of their own validation suite.

Even aside from obvious issues this raises for safety-critical systems, the potential for loss of correctness might reasonably be expected to hinder the adoption of GI in many application areas. For these reasons, it was suggested by White [27] that some variant of formal model-checking be integrated into the optimization procedure.

Another significant issue is that GI often requires a degree of ‘human-in-the-loop’. One recent approach which has the potential to reduce human intervention is the ‘grow and graft’ (termed GGGI) of Harman et al. [11], in which base functionality is evolved via GP (the ‘growing’ phase) and then transplanted (‘grafting’) into a pre-existing host program. Essentially, the ‘grafting‘ consists of replacing a node of the syntax tree with a function call. A case study is given which grows an invocation of the Google Translate API and grafts it into the Pidgin instant messaging system, the latter being a 200 KLoC C/C++ program.

Any attendant necessity for human validation also robs GI of some of the automation that initially motivated it. It might be hoped that GI would be easier for the untrained end-user to apply “off the shelf” than GP, since function and terminal sets could in principle be obtained from the host program. In current practice, a higher degree of human intervention is typically required: e.g. in Langdon and Harman [14], it was necessary to provide ‘match templates’ and perform profiling in order to constrain the space of possible mutations. Since the methods presented here are constrained by the type system, no such configuration activity is necessary.

A third issue (somewhat shared with plain GP) is the trade-off between modularity, expressiveness and readability. It is well-known that GP tends to suffer from bloat and also poor module induction [3]. Insofar as these properties are inherited by GI, there is clearly the potential to degrade the readability of the source program. This effect can be addressed at the potential expense of expressiveness by limiting the permissible mutation operators and/or the scope within which variables can be referenced [14, 28]. By contrast, the technique we describe in this article is intrinsically modular: a direct mapping (a wrapper or adaptor function) is generated between types, which is always representable as a subroutine call. As such, our technique improves upon the state of the art by providing a more adaptable ‘grafting’ phase for ‘grow and graft’ GI, which nonetheless leads to human-readable end results.

### Program Synthesis

There is of course a very large body of pre-existing work on formal approaches to program synthesis. Building on work by Summers [29], there has been much research under the umbrella term of inductive programming [30]. Initial approaches essentially performed symbolic regression and avoided search (stochastic or otherwise) via inductive inference followed by a semantics-preserving construction. This was made possible by two means: a judiciously-chosen set of input–output pairs and a restricted space of programs. More recently, Jha et al. [31] give a method (which they term ‘Iterative Synthesis’) for the construction of loop-free programs from input–output pairs, driven by a SMT solver. The PROSPECTOR system of Mandelin et al. [32] performs type conversion via a collection of operations obtained from existing source code. Perelman et al. [33] and Givero et al. [34] both provide code-completion assistants via type-assisted ranking of API calls for the completion of partial code fragments. In terms of hybridizations between formal and generative techniques, the IGOR II system of Katayama [35] employs an approach termed ‘analytically-generate-and-test’, in order to synthesise programs from input–output pairs.

Of greatest relevance to this article is Djinn [36], which uses a decision procedure to automatically construct functions that map between ADTs in the Haskell language. Since this decision procedure is based on proof search in the intuitionistic propositional calculus [37], Djinn will always find such a function if one exists, or else terminate after constructing an implicit proof of non-existence. However, Djinn does not work with recursive types, since there is no termination guarantee in this case. In the following section, we present our algorithm, which extends Djinn’s approach to deal with a family of recursive types. As a result, our approach is able to generate proofs that Djinn cannot, as described in detail in the following section and illustrated in detail in Sect. 3 and in the case study of Sect. 6, where it is used to synthesize a mapping between two recursively-defined list variants.

### Type Isomorphism

Two types are said to be isomorphic iff. they contain the same information, that is, a pair of mutually inverse conversion functions can be defined between them. Considering types up to isomorphism allows us to ignore superfluous details in data representation. For example, functions that solely disagree in their argument order have isomorphic types.

Describing when two types are isomorphic (and witnessing the isomorphism with conversion functions) is an active research area. An important technique is to associate an equational theory to the types of the language such that the equality $A = B$ follows from the axioms of the theory precisely when the types *A* and *B* are isomorphic. Since recursive types can be characterized as fixed-points of equations, a similar association will prove useful in Sect. 3.2. Bruce and Longo [38] showed that the simply typed lambda calculus admits such theories with a single axiom. The result was subsequently extended to the lambda calculus with surjective pairing [39], and later to several different combinations of type constructors.

Among programming languages, ML’s type system has been the main subject of research. Type isomorphism algorithms have found important applications in API search engines. The idea of using types as keys in a database was first proposed by Rittri [40]. Di Cosmo [41] gave an efficient algorithm for deciding type isomorphism in ML-style polymorphic type assignment frameworks, and applied it to API search engines.

On the logic side, type isomorphism corresponds to the existence of proofs $A \vdash B$ and $B \vdash A$ that reduce to the axiom rule when cut against each other. Finding such proofs is possible using the Proof Search paradigm presented in the article. One should note, however, that some of our grow-and-graft applications are not direct instances of the type isomorphism problem. For example, many types used in the case studies of Sect. 6 fail to be isomorphic, yet one can still derive useful injections and partial inverses between them.

## The Algorithm

### Algebraic Data Types

We now describe our approach, the concrete implementation of which we call PolyFunic. First of all, we associate an algebraic equation to each type. The latter stages of our algorithm (the so-called Proof Search phase) will operate directly on this algebraic representation. In the following, we distinguish between *data types* and *function types* (exponentials). Each data type is associated with a finite set of constructors, and the constructor arguments can be accessed positionally during pattern matching. There are three fundamental constructions for defining new types based on existing ones *S* and *T*:

1. Disjoint union: a data type with two constructors, one having an argument of type *S*, the other an argument of type *T*. Denoted $S+T$.
2. Cartesian product: a data type whose only constructor is a pairing operation with two arguments, having types *S* and *T* respectively. Denoted $S \times T$.
3. Exponentiation: the type whose inhabitants are functions from *S* to *T*. Denoted $T^S$.

With this notational convention, any data type can equivalently be characterized using an algebraic equation given in terms of its constructors. Let *S* be a data type with two constructors, $c_1{:}\,A_1 \times A_2 \rightarrow T$ and $c_2{:}\,A_3 \rightarrow T$. Then the corresponding algebraic equation is

$$\begin{aligned} S = A1 \times A2 + A3 \end{aligned}$$

Generally: take a data type *T* and denote its set of constructors by $C_T$. For $c \in C_T$, let the list of arguments of *c* be given by $A_c$ (the arguments themselves may be function types as well). The algebraic equation for the type *T* is then given by

1 $$\begin{aligned} T = \sum _{c \in C_T} \prod _{a \in A_c} a \end{aligned}$$

Constructors without any arguments are traditionally denoted by 1 in the algebraic equation, since such constructors arise from the vacuous product. To illustrate this, first consider a data type describing a 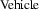: two constructors, 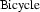 which takes no arguments, and 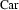 which takes two arguments, the colour and the plate number. The Scala declaration of this type can be seen on Listing 2. The corresponding algebraic equation is simply

2 $$\begin{aligned} {\text {Vehicle}} = 1 + {\text {Color}} \times {\text {String}} \end{aligned}$$

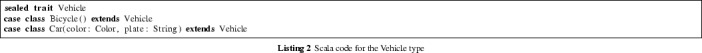


Now consider the data type of *natural numbers*. In Scala, this can be described as per the 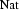 data type of Listing 3. The type has two constructors, i.e. 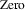, and the 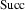 constructor which gives the successor of its argument, another 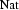. The argument of the constructor 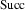 is another 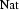. The associated algebraic equation is therefore

3 $$\begin{aligned} {\text {Nat}} = 1 + {\text {Nat}} \end{aligned}$$

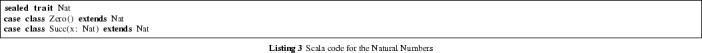


Unlike Eq. 2, Eq. 3 is “recursive” since the term ${\text {Nat}}$ occurs on both sides. Normally, the Proof Search is non-terminating in the presence of such self-reference. Fortunately, in many cases, it is possible to eliminate the self-reference using a technique from category theory.

### Categories and Functors

To explain the elimination of self-reference, it is necessary to briefly introduce some category-theoretic notions. We will attempt to convey the essential concepts without undue technical detail. Category theory has served as a formidably-powerful unifying mechanism in mathematics, but also has widespread applications in software engineering [42], where it provides principled constructions for many kinds of software artifacts. The reader who desires a more in-depth introduction is referred to Pierce [43] or Bird and de Moor [20].

Formally, a *category* is formed by a collection of objects, and an associated collection of morphisms, mapping objects to objects. It is required that there is an identity morphism, i.e. $id{:}\,X \rightarrow X$ for all objects *X* and that morphisms compose, i.e. for objects *A*, *B*, *C*, given morphisms $f{:}\,A \rightarrow B$ and $g{:}\,B \rightarrow C$, a composite morphism $g \circ f{:}\,A \rightarrow C$ can be constructed. The most well-known category is the category having all sets as objects and all functions as morphisms. Up to technical considerations, it is possible to consider a category having Scala types as objects, and Scala functions as morphisms.

A *functor* *F* is a mapping between categories: it associates to every object *A* of the source category an image object *F*[*A*] of the target category, and to every morphism $m{:}\,A\rightarrow B$ in the source category an image morphism $map_F[m]$ in the target category. The image of an identity morphism is required to be an identity morphism.

Intuitively, we can imagine a functor *F* as a generic container.^1^ The notation *F*[*T*] denotes a container *F* parametrised by the type variable *T*. The container *F* can be then instantiated with any of a family of types, e.g. the 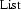 data type is a functor, which can be instantiated as a list of integers 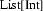, a list of strings 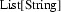 and so on.

Functors must provide a structure-preserving *map* operation which, when provided with *F*[*T*] and a means of conversion between the type *T* and the type *U*, yields an isomorphic container of type *F*[*U*]. For example, the *map* operation of the 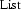 functor preserves the order of the elements:

$$\begin{aligned} {\text {map}}_{{\text {List}}}\left( f, [e_1, e_2, \ldots e_n]\right) \mapsto \left[ f(e_1), f(e_2), \ldots f(e_n)\right] . \end{aligned}$$

The concepts of strong typing and type constructors are of course entirely familiar within the formal methods community. The GP community has become familiar with the use of types to constrain the search space of programs [44] and the notion of a type constructor has also become a familiar concept via its incorporation into object-oriented languages. The same holds for parametric polymorphism, known as “generic types”, or sometimes simply “generics” in the object-oriented context. In terms of foundational computer science, many typed programming languages (e.g. the simply-typed lambda-calculus [45] or intuitionistic type theory [46]) give rise to corresponding categories: the objects are types and morphisms are functions between types.

The following steps (see Bird and de Moor [20] for further detail) allow us to eliminate self-reference from a recursive type *T* when our goal is to find a mapping to another type *R*:

1. Define a functor $B_T$, called the *base functor* associated with the type *T*.
2. Every function $f{:}\,B_T[R] \rightarrow R$ naturally gives rise to a function, called a *catamorphism*, $\mathrm {cata}(f){:}\,T \rightarrow R$.
3. The proof search will look for a proof of the non-recursive sequent^2^ $B_T[R] \vdash R$, yielding a function of type $B_T[R] \rightarrow R$. By constructing the corresponding catamorphism, we obtain a function of type $T \rightarrow R$.

Unless certain technical conditions apply [20], the base functor for *T* can be obtained from the right-hand side of the algebraic equation of *T* by replacing the recursive occurrences of type constructors with an additional type parameter *R*. For example, in the case of *Nat*, the base functor is defined by the equation

4 $$\begin{aligned} B_{{\text {Nat}}}[R] = 1 + R \end{aligned}$$

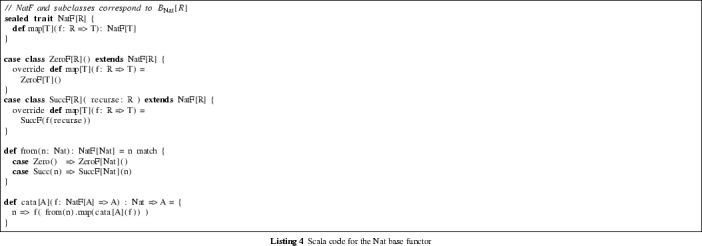


The ultimate goal of PolyFunic is transforming a given ADT *T* (or a set of ADTs $\varGamma$) to some other ADT *U*. Equipped with the base functor and the *cata* function, we can reduce the associated proof search for $T \vdash U$ to a search for a proof of $B_{T}[U] \vdash U$. Notice that this latter sequent does not contain the recursive data type *T*, and is thus amenable to Djinn-style proof search.

This simplification is made possible by virtue of the fact that the fixed point of the functor $B_{{\text {T}}}$ is isomorphic to the type 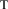. Naturally, the fixed point *X* of a functor *F* is defined by the property^3^ $F[X] = X$. The Scala code corresponding to these constructions can be seen in Listing 4. In general, the code for $B_{T}[R]$ can be synthesized from the data type definition of *T* (e.g. as obtained by Scala’s reflection mechanism [47]). The 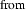 function can be derived for any data type by the trivial bijection between the constructors of *T* and $B_{T}[R]$. The version of *cata* given is a specific case of the general parametric *cata* function, which can operate on any base functor.

### The Search Process

A sequent is of the form $\varGamma \vdash \varDelta$, where $\varGamma$ and $\varDelta$ are comma-separated lists of Scala ADTs.^4^ The intended reading of the sequent is that “given instances of *all* types in $\varGamma$, we can construct an instance of *some* type in $\varDelta$”. Therefore, commas on the left of the $\vdash$ symbol correspond to products, on the right of the $\vdash$ to sums. Many readers will be familiar with the concept of *pattern matching* in functional languages, which is essentially an ordered sequence of condition-action rules mapping from patterns (as described by type constructors) to executable code.

In our proof search, the types are manipulated using *left* and *right* rules of inference derived mechanically from the definition of the corresponding data types. The search starts from the root of the tree (i.e. the rules are meant to be read from bottom to top): it is sufficient to prove the sequents above the horizontal line to conclude the sequent below the horizontal line. Left rules corresponds to pattern-matching on the type, while right rules corresponds to invocations of a constructor of the type. The left rule for a general ADT *T* can be seen below: every branch corresponds to a constructor *c* of *T*, and $A_c$ is the corresponding set of constructor arguments. A single branch corresponds to one possible result of pattern matching.
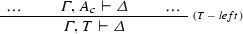


The right rule for a general ADT *T* is similarly derived. In this case, branches correspond to elements *P* of the Cartesian product $\prod _{c \in C} A_c$, thus accounting for all the possible ways of supplying arguments to the constructors:
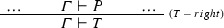


There are separate left and right rules for dealing with function types, given below: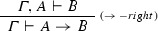 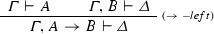


In order to preserve termination in the case of recursive types, the application of left rules has to be forbidden. Instead of left rules, the synthetic rule seen below must be used. This synthetic rule is derived from the catamorphic substitution technique explained in Sect. 3.2, and corresponds to an invocation of the parametric 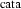 function presented there.
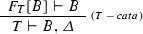


By repeated applications of these rules of inference, a branching *proof tree* is constructed. A branch of the search is terminated when reaching an *axiom* (a sequent such that $\varGamma \cap \varDelta$ is non-empty), or when no further rules are applicable. A proof tree is valid if and only if all the branches terminate in axioms. A sequent with a valid proof tree is said to be *proven*, the corresponding proof tree being the *proof*.

### Proof Search and Code Generation

As described in the previous section, proofs in the sequent calculus are constructed in a bottom-up fashion, starting from the conclusion and repeatedly applying inference rules until axioms are reached. At every stage of the search, only finitely-many rules are available. PolyFunic uses a simple deterministic search procedure with backtracking, which is able to exhaust all possible proofs of a given sequent. An overview of the usual techniques of proof search is given in Paulson [48]. Using a proof of the sequent $A \vdash B$, one can derive Scala functions of signature $f{:}\,A \rightarrow B$. A single proof always corresponds to a single^5^ such function and the correspondence between left rules/pattern matching and right rules/constructor invocations can be used to procedurally generate the corresponding transformation. Having factored out recursion as described above, the generating procedure is precisely that implemented by Djinn [36].

#### Example

As a simple worked example of the search process and corresponding code generation, consider the data types 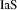 and 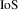 defined in Listing 5.


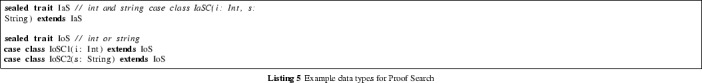


For these types, the general construction outlined above gives the following left and right rules of inference: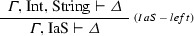 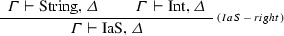 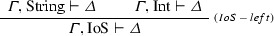 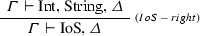


Clearly, an instance of the class 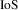 can be extracted from an instance of the class 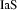 in two ways: one can extract either the 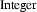 or the 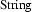. However, it is not possible to extract an 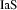 instance from an 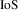 instance: one would need to create either the 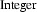 or the 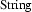 (neither of which are ADTs) from scratch. These results are easily established via Proof Search. We try to prove the sequents $IaS \vdash IoS$ and $IoS \vdash IaS$ respectively. In the first case, the resulting proof will generate the code for the two possible extraction functions. In the second case, the search will fail when the pool of possible rules is exhausted. The search for $IaS \vdash IoS$ yields the following proof (the LaTeX code for the proof trees was generated by PolyFunic): 
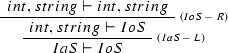


There are two Scala functions corresponding to this proof, one for each type that occurs on both sides of the axiom, as follows:
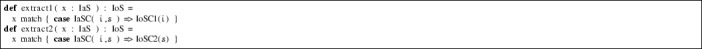


Here is an invalid proof tree, corresponding to a failed search for a proof of the false implication $IoS \vdash IaS$: 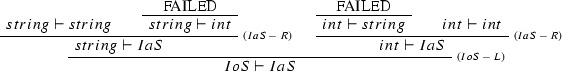


### Limitations

The algorithm presented above is a simple extension of Proof Search in the LJT calculus to deal with recursively defined types. While every valid proof tree of conclusion $A \vdash B$ corresponds to a Scala function of type $A \rightarrow B$, it should be clear that the converse fails in the presence of recursive types. Finding an explicit counterexample is left as an exercise to the reader. As such, implementing some of the type isomorphism approaches presented in Sect. 1 could be a useful—if somewhat ad-hoc—way to further increase the capabilities of the algorithm.

Notice also that the FAILED branches correspond to the cases where either an 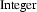 or a 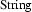 would have to be generated from scratch, which suggests an opportunity for hybridization with some kind of generative technique, e.g. traditional GP. In the following sections, we present two case studies that reveal how the Proof Search algorithm can be used in the ‘grafting’ phase of ‘grow and graft’ GI.

## Implementation

PolyFunic is designed to extend and replace the grafting phase of GGGP. As such, the output of the growing phase is assumed to be available in the form of a Scala module (class file). The runtime reflection facilities of the language are used to extract the data type declarations and function definitions from the module, which works even if the source code of the grown function is not available.

The other required input is the host program. Since PolyFunic emits Scala code, the source code of the host program has to be available. However, there are no theoretical obstructions that would prevent future version of the tool from providing bytecode output, thus making the source code unnecessary.

### Restrictions

PolyFunic ignores data types that do not confirm to the following rules:

1. The base class *T* has the 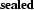 flag.
2. All proper subclasses of *T* have the 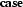 flag.
3. All subclasses extend *T* directly (i.e. no nested chains $T_2 \subseteq T_1 \subseteq T$).

These rules are required because algebraic data types (and by extension, the emitted code) have to support pattern matching. If the conditions are violated, pattern matching could fail to work in the expected manner. In fact, the Scala compiler statically forbids pattern matching unless Rule 2 is satisfied, although it allows partial pattern matches that may result in runtime errors if Rule 2 is satisfied but the other rules are violated.

The restriction is harmless in Scala, but it may cause problems once PolyFunic is extended to work with other languages. For example, Java has no analogues of the 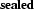 or 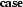 keywords, so new ways of detecting suitable data types will have to be devised. In the worst case, one could always fall back to manual hints and annotations.

### Usage

PolyFunic is designed to be used as a library, integrated with other tools into some GGGP toolchain. The three phases (extraction and grafting, proof search and code generation) are implemented separately and independently. At the time of writing, two code generators are available: one emits executable Scala code based on a proof tree, the other emits LaTeX for pretty-printing proof trees.^6^ Once the input and output modules are designated, the three phases can be executed without human intervention. The implementation achieves good performance by using a functional variant of backtracking: the proof search produces a lazily-evaluated stream of proof trees until the caller finds one acceptable and terminates the process.

### Planned Changes

As noted above, the current PolyFunic implementation uses runtime reflection. Compile time reflection (macros) [49] would allow finer-grained inspection of class declarations, and interfacing with the compiler would allow us to treat extraction, grafting and code generation in a simple, uniform manner. Indeed, the macro approach proved to be extremely versatile in another application of Proof Search to Software Engineering, the Proofbox Dependency Injection container.^7^ As such, it is anticipated that PolyFunic will be extended to use macro-based reflection in the future.

## Case Study: Intelligent Grafting

A fundamental issue in the grafting phase of ‘grow and graft‘ is *type discrepancy*: we want our grown programs to work with inputs on which the desired operations are easily expressed and efficiently implemented: the data structures optimal for the grow phase may be different from the ones used in the host program.

Consider a function, grown in isolation, for computing the Body Mass Index^8^ with type signature $bmi {:}\,{\text {Weight}}\times {\text {Height}}\rightarrow {\text {BMI}}$. Grafting such a function to a host program seems like a simple matter: the possible graft points are the ones in which a variable of type ${\text {Weight}}$ and a variable of type ${\text {Height}}$ are both in scope.

Unfortunately, the useful graft points in a real application are extremely unlikely to be in such scopes. In object-oriented code, properties are not always directly available: in some stages of execution, ${\text {Weight}}$ and ${\text {Height}}$ may appear as member fields of a ${\text {Person}}$ object, while in other stages, only the ${\text {Person}}$’s name is in scope, the actual data being stored in a database, and accessible indirectly through a function such as 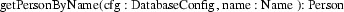.

As stated in the introduction, the ‘grow and graft‘ strategy is intended to be usable without specialist knowledge of the target system [11]. This cannot be achieved unless the grafting procedure can identify valid grafting points without human intervention, while automatically generating the necessary boilerplate code for the appropriate state of execution (extraction methods for member fields,query calls for databases, etc.).

Duplication of effort (evolving a separate function for each different graft point) cannot solve this problem without significant performance and correctness trade-offs. For example, Body Mass Index depends on height and weight only, so a grown program with signature $bmi {:}\,{\text {DatabaseConfig}}\times {\text {Name}}\rightarrow {\text {BMI}}$ is a priori less likely to be correct than one with signature $bmi {:}\,{\text {Weight}}\times {\text {Height}}\rightarrow BMI$. Even if both functions pass the same test cases, the former could still depend on the database configuration (or something more extreme, like the eye color field retrieved from the database) in an unforeseeable manner.

PolyFunic can identify eligible graft points by searching for separate proofs of ${\varGamma _p \vdash {\text {Weight}}}$ and $\varGamma _p \vdash {\text {Height}}$ at each candidate graft point *p*. The symbol $\varGamma _p$ denotes the set of variables in scope at the given graft point. If both proofs are successful, then it is possible to extract ${\text {Height}}$ and ${\text {Weight}}$ objects from the variables in scope, so the grafting can proceed. As detailed in Sect. 3.3, the necessary boilerplate can be derived from the proof trees found by the search procedure.

PolyFunic is able to find non-trivial scopes eligible for grafting, and can automatically generate the boilerplate code necessary for the grafting to proceed. This functionality was tested on the code of Listing 6. The software correctly identified the designated graft point as eligible, even though no explicit variables of type ${\text {Weight}}$ or ${\text {Height}}$ are in scope. It generated and inserted boilerplate (programmed against a database query interface) that makes the grafting possible. This avoids the need for duplication of effort, and preserves tight correctness guarantees achieved by a good choice of data structures in the grow phase. PolyFunic finds and evaluates all potential graft points in less than a second for the code considered above.^9^ Being type-aware significantly reduces the size of the search space for possible insertion points and for identifying the shared parameters between the donor and the host. In particular, insertion locations that would normally result in an expensive failed compilation are guaranteed to be skipped.
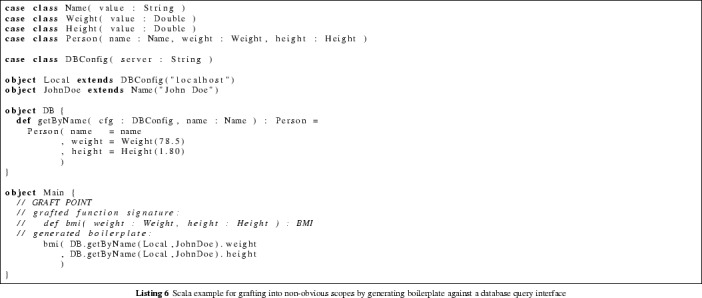


## Case Study: Asymptotic Improvement

The grafting phase of ‘grow-and-graft’ presents some other challenges as well. For example, if the desired functionality is grown in sufficient isolation, the grafting will never succeed, since the grown subroutine will be unaware of the types/data structures used by the parent program. Even if we restrict the data structures to ADTs, there are many inequivalent ways of implementing e.g. a list as an ADT. This is a serious concern, especially since different implementations place different constraints on the grown programs.

PolyFunic can use proof search to automatically identify which data types of a host program can be substituted for data types of the grown program, even if the desired functionality was grown in isolation, and the data structures don’t share a common interface. This effectively reduces the number of useless graft operations by ruling out grafts between provably incompatible types, while increasing the number of potentially useful graft operations: PolyFunic will create mappings between data structures that are implemented differently, but are otherwise compatible.

This allows exploration of the various trade-offs between different implementations of a data structure. For example, the ubiquitous heap data structures provide a variety of operations such as 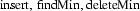 etc. with many possible concrete implementations (e.g.$\tilde{\mathrm{b}}$inary, binomial, Fibonacci) each exhibiting a different trade-off in asymptotic performance [50].

Without PolyFunic, graft operations would not be able to satisfy the intended semantics if the types used by the parent program and the grown program are not compatible at the level of interfaces.^10^ Fortunately, the PolyFunic implementation can automatically extract the data type definitions from the source code of the programs, and identify which ADTs are compatible (by attempting to prove $T_i \vdash T_j$ for every ADT $T_n$). As seen in Sect. 3.3, the successful proofs give rise to appropriate type conversion functions. If the data types are recursively defined, it is necessary to factor out recursion using the catamorphism approach described in Sect. 3.3.

### ConsList and SnocList

As a concrete instance of this problem, take the two ways of building linked-lists as ADTs: the constructors correspond either to prepending or appending an element, yielding what are traditionally called 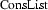 and 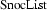 respectively and are given in Listing 7. The two types are, of course, isomorphic, and PolyFunic is able to derive conversion functions between them.
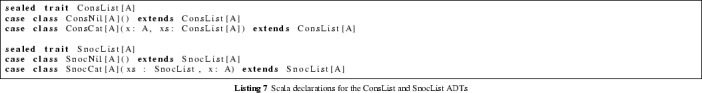


When asked to prove $ConsList \vdash SnocList$, PolyFunic returns the following proof tree (among others). For reasons of space, 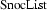 and 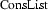 are abbreviated by *S* and *C* respectively in the proof below.
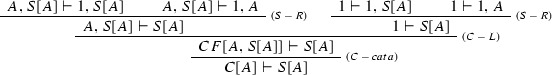


This proof corresponds to the obvious conversion function between 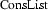 and 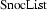
—in the specific case of lists, the 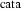 function corresponds to the ubiquitous higher-order *fold* function, which turns iteration over the list into recursion. In the case of 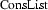, *fold* acts on the constructors by replacing 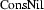 with a value 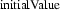 and 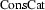 with a binary operation 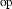. Hence, for the list [1, 2] given by 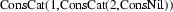, *fold* yields *op*(1, *op*(2, *initialValue*)). The conversion function derived by PolyFunic is equivalent to a fold with 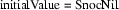 and 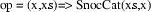. In the program of Listing 8, the *initialValue* and the *op* correspond to the branches of the pattern matching.
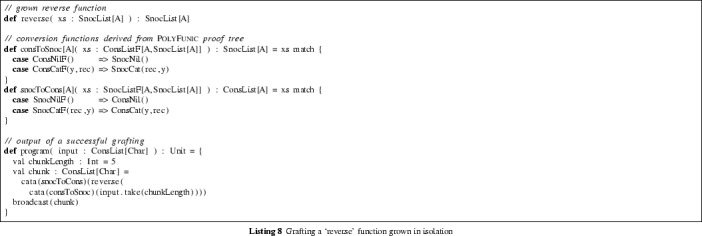


### Asymptotic Efficiency Improvement

As discussed above, the naïve application of GI suffers from a lack of guarantees: most notably of correctness, but also of efficiency. Even when the latter is an explicit objective, it is typically the case that this objective is obtained on some training set, in anticipation that this will generalize to the scenario of end-use. In closing, we describe a transformation that is not only semantics-preserving, but which also leads to an asymptotic improvement in efficiency.

Many immutable data structures, such as 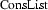 above, or regular Java™’s 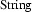 class, have poor asymptotic performance on common functionality such as repeated concatenation or even the 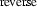 function (for a broader discussion of this phenomenon the reader is referred to [51]). This motivates alternative data representations, such as the infamous 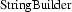
s, and the difference lists presented below.

A difference list is a functional data structure introduced by Hughes [51], which supports constant-time concatenation operations.^11^ Every difference list *f* represents an underlying linked list $L_f$. The difference list itself is implemented as a function $f{:}\,ConsList[A] \rightarrow ConsList[A]$ which works by prepending $L_f$ to its argument. As such, only some functions with that signature represent linked lists, i.e. the types 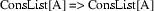 and 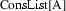 are not isomorphic. The Scala declaration for a difference list ADT is the following:
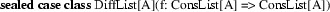


Concatenation of difference lists is simply composition of their associated functions. As such, a grown code fragment which uses difference lists for a function that requires many concatenation operations will be asymptotically superior to one that works directly on linked lists. Such operations are required even for relatively common functionality such as *reverse*, see [51]. As discussed at the beginning of this section, there is a need for grown functionality to use types that achieve good asymptotic performance, but this is problematic since the data structures of the host program are fixed. The grafting phase must therefore accommodate this type discrepancy. Given the declaration of 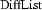 above, PolyFunic’s proof search is able to derive the proof for converting between the 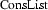 and 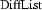 data structures, which allows the grown functionality to work on the latter ADT, even though the host program uses the former, thus allowing for asymptotically superior performance.

## Conclusion

In this article, we presented a deterministic search method for deriving semantics-preserving transformations via proof search in the sequent calculus and use of the correspondence between Scala case classes and algebraic data types. We demonstrated that this approach can be used to address important shortcomings of Genetic Improvement and more specifically, the recently developed ‘grow and graft’ Genetic Improvement [11].

The main limitations of PolyFunic are external: as discussed in Sect. 1, the software preserves only the semantic content that is made explicit in the type declarations. This limits applicability to programs which reveal significant amounts of semantic information in their type signatures. Fortunately, modern object-oriented applications developed in Scala invariably belong to this class. Another limitation is that the method relies on the powerful type system and reflection capabilities of the Scala programming language. Writing an implementation of the algorithm used in PolyFunic that works with more widely-used languages with simpler type systems (such as C and Java) is expected to be difficult, although with Java this issue can be circumvented to some degree by the bytecode-compatibility between Java and any generated Scala code.

In its present form, the proof search can sometimes stall for want of an instance of a non-algebraic data type (e.g. an integer argument determining the size of an array). There are opportunities to overcome this problem by hybridizing with generative techniques. Another possible direction of future work is extending PolyFunic to automatically harvest and reassemble code fragments taken from the open-source code bases available on the Internet.

Our case study shows that it is possible to transform invocations of the list data type to yield an asymptotic efficiency improvement. We intend to extend this application of PolyFunic to a variety of other data types such as various heaps, binary trees and finger trees [52].

## Footnotes

1 We freely skip technical considerations that are irrelevant for our purposes. For example, the notion that’s really described here is that of an *endofunctor* in the category of Scala types.

2 Sequent notation is described thoroughly in Sect. 3.3.

3 It is possible to express the fixed-point construction internally in Scala, using a type-level operation commonly referred to as *Mu* or *Fix*.

4 Proof-theoretically, this corresponds to the sequent calculus for propositional logic.

5 To be precise, up to a minor ambiguity handled by explicitly identifying the type on which the axiom is invoked.

6 As mentioned earlier, the proof trees in the present article were generated directly by this backend.

7 See https://github.com/zaklogician/Proofbox/.

8 body weight in kilograms divided by the square of the height in meters.

9 Test environment: Intel Core™ i7-4712MQ CPU @ 2.3 GHz and 8 GB RAM.

10 This happens often when a program implements its own container types for efficiency purposes.

11 For comparison: concatenation is linear time for the two linked lists as algebraic data types introduced in the previous section.
